# Activation of Orexin System Stimulates CaMKII Expression

**DOI:** 10.3389/fphys.2021.698185

**Published:** 2021-07-01

**Authors:** Yuanyuan Fan, Enshe Jiang, Huanjia Gao, Jeremy Bigalke, Bojun Chen, Chunxiu Yu, Qinghui Chen, Zhiying Shan

**Affiliations:** ^1^Department of Kinesiology and Integrative Physiology, Michigan Technological University, Houghton, MI, United States; ^2^School of Life Sciences, Henan University, Kaifeng, China; ^3^Institute of Nursing and Health, Henan University, Kaifeng, China; ^4^The Second Clinical College, Guangzhou University of Chinese Medicine, Guangzhou, China; ^5^Department of Emergency, The Second Affiliated Hospital, Guangzhou University of Chinese Medicine, Guangzhou, China; ^6^Health Research Institute, Michigan Technological University, Houghton, MI, United States; ^7^Department of Biological Sciences, Michigan Technological University, Houghton, MI, United States

**Keywords:** CaMKII, orexin-A, paraventricular nucleus, sympathetic nerve activity, blood pressure

## Abstract

Hyperactivity of the orexin system within the paraventricular nucleus (PVN) has been shown to contribute to increased sympathetic nerve activity (SNA) and blood pressure (BP) in rodent animals. However, the underlying molecular mechanisms remain unclear. Here, we test the hypothesis that orexin system activation stimulates calcium/calmodulin-dependent kinase II (CaMKII) expression and activation, and stimulation of CaMKII expressing PVN neurons increases SNA and BP. Real-time PCR and/or western blot were carried out to test the effect of orexin-A administration on CaMKII expression in the PVN of normal Sprague Dawley (SD) rats and orexin receptor 1 (OX1R) expressing PC12 cells. Immunostaining was performed to assess OX1R cellular localization in the PVN of SD rats as well as orexin-A treatment on CaMKII activation in cultured hypothalamic neurons. In vivo sympathetic nerve recordings were employed to test the impact of optogenetic stimulation of CaMKII-expressing PVN neurons on the renal SNA (RSNA) and BP. The results showed that intracerebroventricular injection of orexin-A into the SD rat increases mRNA expression of CaMKII subunits in the PVN. In addition, Orexin-A treatment increases CaMKII expression and its phosphorylation in OX1R-expressing PC12 cells. Furthermore, Orexin-A treatment increases CaMKII activation in cultured hypothalamic neurons from neonatal SD rats. Finally, optogenetic excitation of PVN CaMKII-expressing neurons results in robust increases in RSNA and BP in SD rats. Our results suggest that increased orexin system activity activates CaMKII expression in cardiovascular relevant regions, and this may be relevant to the downstream cardiovascular effects of CaMKII.

## Introduction

Orexin-A and orexin-B are neuronal peptides processed from the same precursor, prepro-orexin, which is produced by a small number of neurons located in the lateral hypothalamus (Meister and Hakansson, 1998; Sakurai et al., 1998). Although originally recognized as regulators of feeding behavior and wakefulness (Sakurai et al., 1998; Sakurai, 2002), orexins are now regarded as multi-functional peptides that coordinate numerous physiological, and subsequently pathophysiological processes (Thannickal et al., 2000; Harris et al., 2005). The actions of orexin neuropeptides are mediated by orexin receptor 1 (OX1R) and orexin receptor 2 (OX2R), two G-protein coupled receptors (Sakurai et al., 1998; Scammell and Winrow, 2011). The two receptors present specificity in their binding affinity to the orexin neuropeptides, with OX1R having a much higher affinity for orexin-A than for orexin-B, whereas the OX2R binds both orexin peptides with a similar affinity (Sakurai et al., 1998). Although orexin-A is only produced by a limited number of neurons within the lateral hypothalamic area, orexin receptors innervate a vast number of brain and spinal cord structures (Antunes et al., 2001), resulting in far-reaching influences of the sequestered orexin-producing neurons. Through their axonal synapses with their receptor-expressing neurons, they convey signals to postsynaptic neurons, effectively regulating their activities.

Recent efforts have been put forth to elucidate the mechanistic involvement of orexins in blood pressure (BP) and cardiovascular regulation. Studies have shown that orexin system activity is increased in the hypothalamic paraventricular nucleus (PVN), and upregulated orexin system activity is implicated in several forms of hypertension, including salt-sensitive and obesity-related hypertension (Zhou et al., 2015, 2019b; Huber et al., 2017; Fan et al., 2018). Furthermore, antagonism or genetic knockdown of the orexin receptors results in attenuated BP in spontaneously hypertensive rats and obesity-associated hypertensive rats (Li et al., 2013). Despite the clear influence of orexin-A on cardiovascular health and sympathetic nerve activity (SNA), the detailed mechanisms mediating the effect of orexin system activity on BP regulation remain elusive.

Ca^2+^/calmodulin-dependent protein kinase II (CaMKII) is a key protein kinase that is regulated by the Ca^2+^/calmodulin complex (Kool et al., 2019). This multi-faceted complex has been shown to coordinate many signaling cascades which further regulate numerous physiological functions including learning and memory (Kool et al., 2019). However, more recently, it was observed that inhibition of hypothalamic CaMKII reduces elevated BP and sympathetic nerve discharges in hypertension (Li et al., 2017). These findings suggest that overactivation of CaMKII in cardiovascular relevant regions of the brain, such as the PVN, contributes to hypertension development, and maintenance (Zhuang et al., 2016; Li et al., 2017; Zhou et al., 2019b). Furthermore, binding of orexin-A with OX1R can increase intracellular calcium influx (Lund et al., 2000; Putula et al., 2014). Interestingly, increased intracellular calcium concentrations also activate CaMKII (Worrell and Frizzell, 1991). This observation, coupled with the emerging role of orexin and CaMKII in the development of hypertension, offers new mechanistic insight into the pathological development of cardiovascular dysfunction.

To this end, we hypothesize that the binding of orexin-A to OX1R will trigger an increase in CaMKII activation and expression. Therefore, we propose that increased orexin system activity will result in increases in both CaMKII expression and its activity in pre-sympathetic PVN neurons, which may lead to molecular underpinnings responsible, at least in part, for increases in SNA and ultimately hypertension development. In this study, we utilize multiple state-of-the-art techniques involving cell cultures, brain tissues and whole animal studies to investigate the role of OX1R activation on CaMKII expression and activation, as well as the downstream effect of CaMKII-neuron specific activation on SNA and BP.

## Materials and Methods

### Animals

Adult Sprague Dawley (SD) rats were purchased from Charles River Laboratories Inc. and housed in the Michigan Technological University (MTU) animal care facility. The rats were maintained at room temperature (20–24°C) with a humidity of 55 ± 10%. Additionally, animals were maintained on a 12 h light/dark cycle, and were given ad libitum access to food and water. The rats were used for intracerebroventricular (ICV) injection, optogenetic manipulation, renal SNA (RSNA), and BP recordings as detailed below. All experimental procedures were approved by the Michigan Technological University Institutional Animal Care and Use Committee.

### ICV Injection of Orexin-A in SD Rats

To determine the effects of orexin-A ICV injection on PVN CaMKII expression, adult male SD rats were divided into two groups (*n* = 6/group) and received ICV injection of either orexin-A (2 nmol) or vehicle control (0.9% NaCl). The detailed procedures for ICV microinjection can be referred to in our previous publication (Huber et al., 2017). Briefly, adult male SD rats (10–12-week-old) were anesthetized with isoflurane (3%), and injected with either orexin-A (2 nmol dissolved in 0.9% NaCl, 4 μl) or saline (0.9% NaCl, 4 μl) into the lateral ventricle using an injection minipump (Micro 4, WPI). The stereotaxic coordinates for ICV injection were as follows: 0.8–0.9 mm behind the bregma; 1.4–1.8 mm away from the midline; and 3.2–3.8 mm below the surface of the brain. After injection, the rats were returned to a clean cage where they naturally woke up and moved freely several minutes later. Three hours following injection, animals were euthanized, their PVN tissues were harvested, and real-time PCR was performed to measure mRNA of two CaMKII subunits, CaMKIIa and CaMKIIb.

### Optogenetic Stimulation of PVN CaMKII-Expressing Neurons and the Measurement RSNA and BP

Male adult SD rats were randomly divided into two groups (*n* = 3 per group) and received AAV-CaMKII-ChR2 (channel- rhodopsin-2)-eYFP or a control vector, AAV-CaMKII-GFP, via bilateral microinjection into the PVN. Four weeks following viral vector microinjection, when transgenes were fully expressed in the PVN, optogenetic stimulation of CaMKII neurons were performed at different intensities and frequencies. BP and RSNA in response to stimulation were recorded at each specific stimulation level. The rats were anesthetized with an intraperitoneal injection containing a mixture of α-chloralose (80 mg kg^–1^) and urethane (800 mg kg^–1^) and the level of anesthesia was assessed by lack of the pedal withdrawal reflex. They were then instrumented with an arterial catheter inserted into the aorta through a femoral artery. The catheter was connected to a pressure transducer to measure arterial BP (ABP). After tracheal cannulation, rats were paralyzed with gallamine triethiodide (25 mg⋅kg^–1^⋅h^–1^ iv) and artificially ventilated with oxygen-enriched room air. After paralysis, anesthesia was monitored by the stability of ABP and heart rate (HR). The supplements equal to 10% of the initial dose were given when needed. End-tidal PCO_2_ was continuously monitored and maintained within normal limits (35 to 40 mmHg) by adjusting ventilation rate (80 to 100 breaths/min) and/or tidal volume (2.0 to 3.0 ml).

For the optogenetic stimulation, an optical fiber (200 μm core diameter, 1.25 mm outer diameter ceramic zirconia ferrule, Precision Fiber Products) was lowered into the PVN (1.2 to 1.6 mm caudal to the bregma, 0.5 mm lateral to the midline, and 7.0 to 7.2 mm ventral to the dura). Optogenetic stimulation (473 nm laser) was conducted and examined at different intensities (1.0, 5.0, and 20.0 mW at 40 Hz, 5 ms pulse width) and frequencies (10, 20, and 40 Hz with 20 mW end output, 5 ms pulse width). Each stimulation occurred in a train of 3 trials, each trial consisted of 10s stimulation on and 30s stimulation off (allowing the BP to recover to the baseline level after each 10s stimulation).

Renal SNA recording and BP measurement were performed as previously described (Gui et al., 2012; Huber et al., 2017; Fan et al., 2018). Briefly, with the use of a left flank incision, a left renal sympathetic nerve bundle was isolated from surrounding tissue and mounted on a stainless-steel wire electrode (outer diameter: 0.127 mm, A-M Systems) and covered with a silicone-based impression material (Coltene, Light Body) to insulate the recording from body fluids. The recorded signal was directed to an alternating current amplifier (P511, Grass Technologies) equipped with half-amplitude filters (bandpass: 100–1,000 Hz) and a 60-Hz notch filter. The processed signal was rectified, integrated (10-ms time constant), and digitized at a frequency of 5,000 Hz using a 1401 Micro3 analog-to-digital converter and Spike2 software (version 7.04, Cambridge Electronic Design, Cambridge, United Kingdom). Background noise was determined by a bolus injection of hexamethonium (30 mg/kg iv), a ganglionic blocker, at the end of the experiment and was subtracted from all integrated values of SNA. The ABP were recorded and measured with the Spike2 software.

### Measurement of CaMKII mRNA Levels With Real-Time PCR in PVN Tissue and PC12-OX1R Cells

Real-time PCR (RT-PCR) was performed to measure mRNA levels of two subunits of CaMKII, CaMKIIa, and CaMKIIb, in the PVN tissues and cell cultures. For the measurement of CaMKII mRNA levels, PC12-OX1R and control PC12 cells were incubated with orexin-A (100 nM) or vehicle control for differing times (3, 6, and 24 h). Cells were then collected and subjected to real-time PCR to test mRNA expression levels of CaMKIIa and CaMKIIb. To assess the role of Ca^2+^ Influx on Orexin-A Effect, PC12-OX1R cells were pretreated with EGTA-AM (10 mM), a membrane-permeable form of the Ca^2+^-chelating agent EGTA, for 1 h and then incubated with 100 nM of orexin-A for 6 h. The PVN tissues or cell cultures were subjected to RNA extraction using the RNeasy Mini Kit (Qiagen, United States). RNA (200–300 ng) was reverse transcribed to cDNA in 20 μl of PCR reaction system using the iScript^TM^ cDNA synthesis kit (Bio-Rad, United States). The cDNA samples were then used as templates and real-time PCR was performed to measure mRNA levels of CaMKIIa (Rn01258147_m1) and CaMKIIb (Rn00572627_m1) using TaqMan primers and probers manufactured by Applied Biosystems (Foster City, CA, United States). The data were normalized to GAPDH (Rn01775763_g1) mRNA for analysis.

### The Effect of Orexin-A Treatment on CaMKII Activation Using Human OX1R Expressing PC12 (PC12-OX1R) Cells by Western Blotting Methods

PC12-OX1R cells were divided into five groups and received treatment with either vehicle control or orexin-A (100 nM) for differing times (10, 30, 60, and 180 Mins). Cells were then collected and subjected to western blot analysis to test protein levels of CaMKII and CaMKII phosphorylation. Protein samples (25 μg) were separated with 10% SDS-polyacrylamide gel electrophoresis, transferred to nitrocellulose membranes, and probed with mouse anti-CaMKIIa (1:200 dilution), mouse anti-CaMKIIb (1:200 dilution), rabbit anti-P-CaMKII (1:200 dilution), or mouse anti-β-actin (1:200 dilution) antibodies (used as a protein loading control). Hybrid protein bands were detected with ECL^TM^ Western Blotting Detection Reagents (Bio-Lab). Protein bands representing CaMKIIa, CaMKIIb, and P-CaMKII were quantified and normalized with the β-actin band using the NIH ImageJ program (Bethesda, MD, United States)^1^.

### OX1R and CaMKII Immunoreactivity in the PVN and Cultured Hypothalamic Neurons

#### Immunofluorescence in PVN Tissue

Male adult SD rats (*n* = 3) were deeply anesthetized with 5% isoflurane. Next, cold phosphate buffer saline (PBS) followed by 4% paraformaldehyde (PFA) in 1×PBS was used to transcardially perfuse the animals. Following perfusion, the brains were removed and kept in 4% PFA overnight at 4°C. Brains were then transferred and kept in 30% sucrose in 1×PBS at 4°C until they sank to the bottom. Brains were cut in 25-μm coronal sections, and the sections containing the PVN area were used to perform co-immunostaining of OX1R with either neuronal nuclei (NeuN, a marker for neurons), glial fibrillary acidic protein (GFAP, a marker for astrocyte), or ionized calcium-binding adaptor molecule 1 (Iba1, a marker for microglia) to investigate in which type of cells OX1R is primarily distributed within the PVN.

The sections (25 μm) were first incubated with PBS containing 0.2% triton-X-100 and 2% horse serum for 1 h, and then were divided into three groups. Next, they were incubated in a cocktail containing goat anti-OX1R antibody (1:50 dilution)/rabbit anti-NeuN (1:50 dilution) (Group 1), goat anti-OX1R (1:50 dilution)/rabbit anti-Iba1 (1:1000 dilution) (Group 2), or rabbit anti-OX1R (1:200 dilution) /mouse anti-GFAP (1:200 dilution) (Group 3) in the PBS for 48–72 h. The brain sections were washed with PBS three times, 10 min each. Afterward, the brain sections were incubated with a mixture of secondary antibodies: Alexa Fluor 488 donkey anti-goat IgG/Alexa Fluor 594 donkey anti-rabbit IgG (Group 1-2), or Alexa Fluor 488 donkey anti-rabbit IgG/Alexa Fluor 594 goat anti-mouse IgG (Group 3). All secondary antibodies were diluted in 1:1000 solution. The brain sections were washed again with PBS three times, 10 min each. Lastly, the sections were mounted in Vectorshield (Vector Labs, Burlingame, CA, United States) and images were taken with a Leica DMIL fluorescence microscope.

#### Primary Neuronal Culture Preparation and Testing

Neuronal cells were isolated from the hypothalamus of 1-day-old SD rats as described previously (Shan et al., 2008). Next, cells were plated in poly-D-lysine precoated 12-well cell culture plates (10^5^ cells/well) in Neurobasal -A medium (Thermo Fisher Scientific) supplemented with 2% B27+ (Thermo Fisher Scientific) and 1% antibiotic-penicillin/streptomycin (Invitrogen). The cell cultures were incubated at 37°C in a 5% CO_2_ incubator for 10–14 days before orexin-A treatment.

After 10–14 days, these neuronal cultures were treated with 1 μM orexin-A or vehicle control for 15 min and fixed with 4% PFA. They were then subjected to immunofluorescent staining against P-CaMKII antibodies followed by a secondary antibody incubation. The P-CaMKII immunoreactivity was observed under a fluorescent microscope and images were taken.

### Reagents and Antibodies

The reagents and antibodies utilized in the current study have been outlined in Table 1. Orexin-A, gentamicin, and EGTA-AM were purchased from Sigma-Aldrich Co. (St. Louis, MO, United States). Goat anti-OX1R antibody was purchased from OriGene Technologies (Rockville, MD, United States); rabbit anti-OX1R antibody was a product of Alomone Labs (Jerusalem, Israel); rabbit anti-NeuN and rabbit anti-GFAP antibodies were products of Cell Signaling Technology (Danvers, MA, United States); rabbit anti-Iba1 antibody was purchased from Wako; Primary antibodies mouse anti-CaMKIIa, mouse anti-CaMKIIb, and mouse anti-P-CaMKII were products of Santa Cruz Biotechnology (Dallas TX, United States). Secondary antibodies including goat anti-rabbit IgG (H+L) Alexa Fluor^®^ 488 and donkey anti-mouse IgG (H+L) Alexa Fluor^®^ 594 were purchased from Thermo Fisher Scientific (Waltham, MA, United States). Real-time PCR primers specific for CaMKIIa and CaMKIIb were purchased from Thermo Fisher Scientific (Waltham, MA, United States). AAV-CaMKII-ChR2-eYFP and AAV-GFP were purchased from the University of North Carolina vector center.

**TABLE 1:** The information of the antibodies used in the study.

**Antibodies**	**Company**	**Catalog number**	**Source**	**Dilutions for WB**	**Dilution for IF**
**Primary antibodies**					
OX1R	Acris	Ap31067pu-n	Goat	/	1:50
OX1R	Alomone labs	AOR-001	Rabbit	/	1:100
NeuN	Cell Signaling	mAb#24307	Rabbit	/	1:50
Iba1	Wako	019-19741	Rabbit	/	1:500
GFAP	Invitrogen	mAb#3670	Mouse	/	1:300
Anti-pCaMKII	Santa cruz	sc-32289	Mouse	1:1000	1:200
CaMKIIa	Santa cruz	sc-13141	Mouse	1:1000	/
CaMKIIb	Santa cruz	sc-376828	Mouse	1:1000	/
β-actin	Santa cruz	sc-47778	Mouse	1:1000	/
**Secondary antibodies**					
Anti-goat 488	Invitrogen	A32814	Donkey	/	1:1000
Anti-rabbit 594	Invitrogen	A-21207	Donkey	/	1:1000
Anti-rabbit 488	Invitrogen	A-21206	Donkey	/	1:1000
Anti-mouse 594	Invitrogen	A-11005	Goat	/	1:1000
Anti-mouse IgG (HRP)	Invitrogen	PA1-28568	Rabbit	1 :2000	/

*WB, western blot; IF, immunofluorescence.*

### Statistical Analysis

All data are presented as the mean ± SEM. Depending on the experiment, statistical analysis was conducted by either independent samples *t*-test or one-way analysis of variance. If statistical significance was observed in the ANOVA analysis, Tukey post-hoc pair-wise multiple comparisons were conducted to assess differences between treatments/conditions. A value of *P* < 0.05 was considered to be statistically significant.

## Results

### Central Administration of Orexin-A Increases mRNA Expression of CaMKII Subunits in the PVN of SD Rats

**FIGURE 1:**
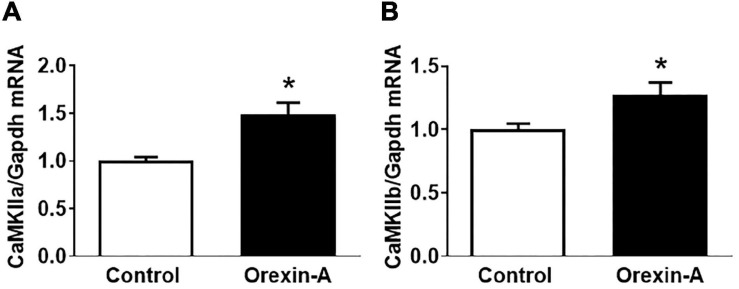
ICV injection of orexin-A increases mRNA expression of CaMKIIa and CamKIIb in the PVN of SD rats. Adult male SD rats received ICV injection of either orexin-A or vehicle control. The rats were euthanized 3 h following microinjection, and PVN tissue was harvested and subjected to real-time PCR to measure mRNA expression of CaMKIIa **(A)** and CaMKIIb **(B)**. The data were normalized to GAPDH mRNA and the average mRNA levels in control rats were assigned an arbitrary unit of 1 (*n* = 5/group). *P* = 0.006 for the CaMKIIa mRNA **(A)** increase and *P* = 0.022 for the CaMKIIb mRNA **(B)** increase. * *P* < 0.05.

Intracerebroventricular injection of orexin-A into the SD rats resulted in significant increases in the mRNA expression of CaMKIIa (1.5-fold) and CaMKIIb (1.3-fold) compared to control saline injection rats (Figure 1).

### OX1R Is Primarily Distributed in Neurons in the PVN of SD Rats

**FIGURE 2:**
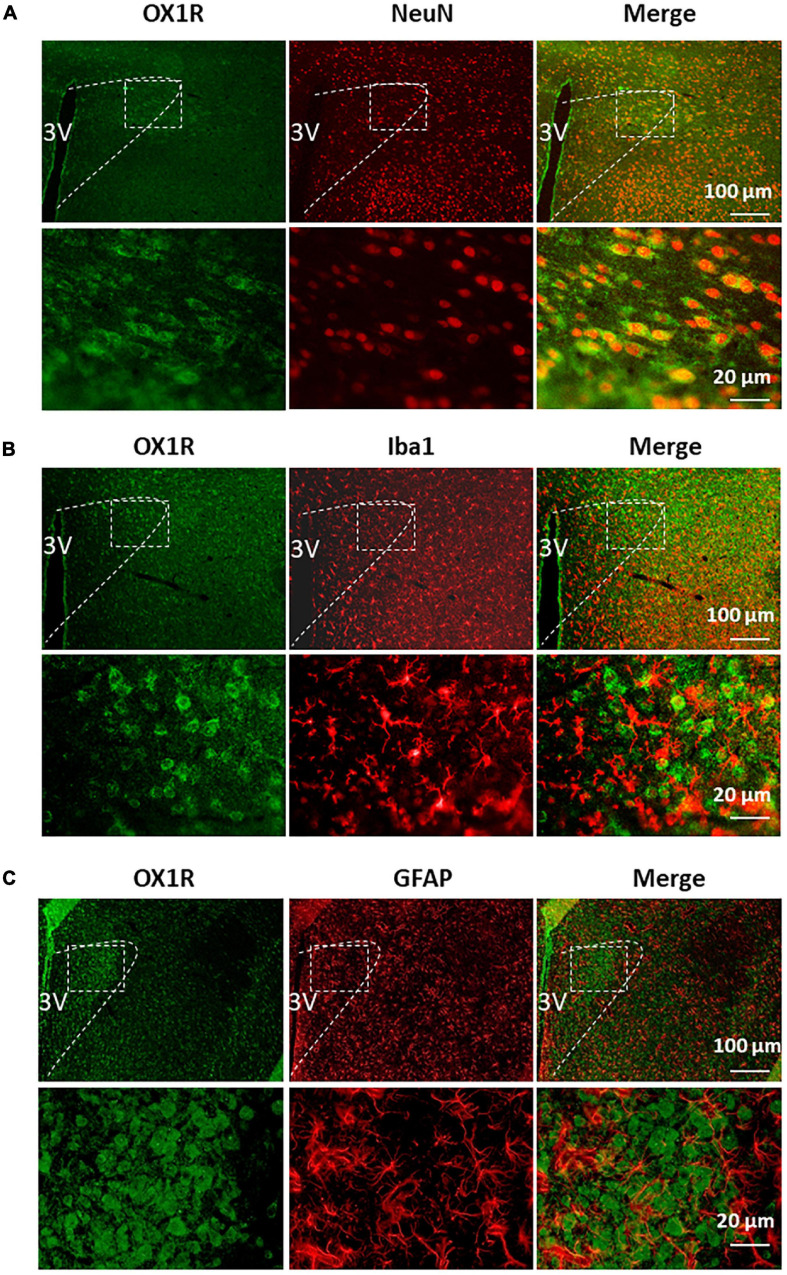
PVN OX1R is primarily expressed in neurons in the SD rats. Representative images showing immunoreactivity of OX1R (green), neuronal nuclei NeuN [red **(A)**], microglial marker Iba1 [red **(B)**], astrocyte marker GFAP [red **(C)**], and merged images in the PVN of SD rat. The area labeled in the dashed rectangle in the upper panel was magnified and showed in the lower panel. The brain coronal sections were taken from 1.8 mm caudal from the bregma. 3V, the third ventricle.

Our previous study demonstrates that OX1R is the primary orexin receptor distributed in the PVN of SD rats (Fan et al., 2018). Given the heterogeneity in cell types within the PVN (neurons, astrocytes, microglia), we sought to assess in which type of cells OX1R is primarily expressed. Co-immunostaining of OX1R with an antibody, either anti- NeuN (a marker for neurons), anti-GFAP (a marker for astrocyte), or anti-Iba1 (a marker for microglia), showed that OX1R is primarily distributed in NeuN-positive cells in the PVN (Figure 2A). There is no co-localization of OX1R with Iba1 (Figure 2B). OX1R immunoreactivity is also observed in some GFAP-positive cells (Figure 2C). These suggest that PVN OX1R are primarily distributed in neurons and some astrocytes.

### Orexin-A Treatment Stimulates CaMKII Expression and Activation in PC12-OX1R Cells

**FIGURE 3:**
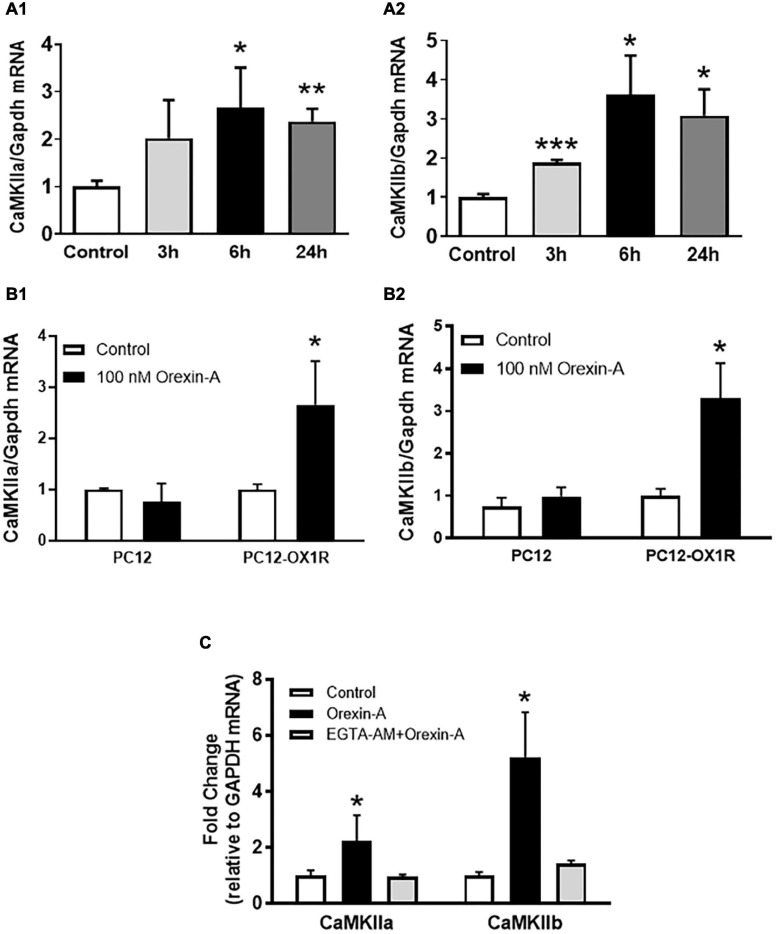
Orexin-A treatment increases mRNA expression of CaMKII subunits in PC12-OX1R cells. **(A)** Orexin-A (100 nM) stimulation induced a time-dependent increase in mRNA expression of CaMKIIa **(A1)** and CaMKIIb **(A2)** subunits in PC12-OX1R cells. **(B)** Orexin-A (100 nM) treatment for 6 h induced increases in mRNA expression of CaMKIIa **(B1)** and CaMKIIb **(B2)** subunits in PC12-OX1R, but not in PC12 cells. **(C)** The effect of orexin-A on CaMKII expression is blocked by EGTA-AM. The data were normalized to GAPDH mRNA. Average control mRNA level was assigned as arbitrary unit 1 (*n* = 6 per group, *P < 0.05, ** *P* < 0.01, *** *P* < 0.001).

Using PC12-OX1R cells, a recombinant human OX1R-expressing neuron-like PC12 cells, we investigated whether OX1R activation can increase CaMKII expression as well as activate CaMKII. The control PC12 cells are derived from the rat adrenal pheochromocytoma and do not express any orexin receptors. Orexin-A treatment resulted in a time-dependent increase in both CaMKIIa and CaMKIIb mRNA expression, with the maximum increase occurring 6 h following orexin-A treatment (CaMKIIa: 2.7-fold; CaMKIIb: 3.6-fold; *P* < 0.05) (Figure 3A) in PC12-OX1R cells. In contrast, orexin-A treatment in PC12 cells for 6 h does not cause any alteration in CaMKIIa and CaMKIIb expression (Figure 3B). The increases in CaMKIIa and CaMKII induced by orexin-A were completely abolished by EGTA-AM (10 mM), a membrane-permeable form of the Ca^2+^-chelating agent EGTA (Figure 3C).

**FIGURE 4:**
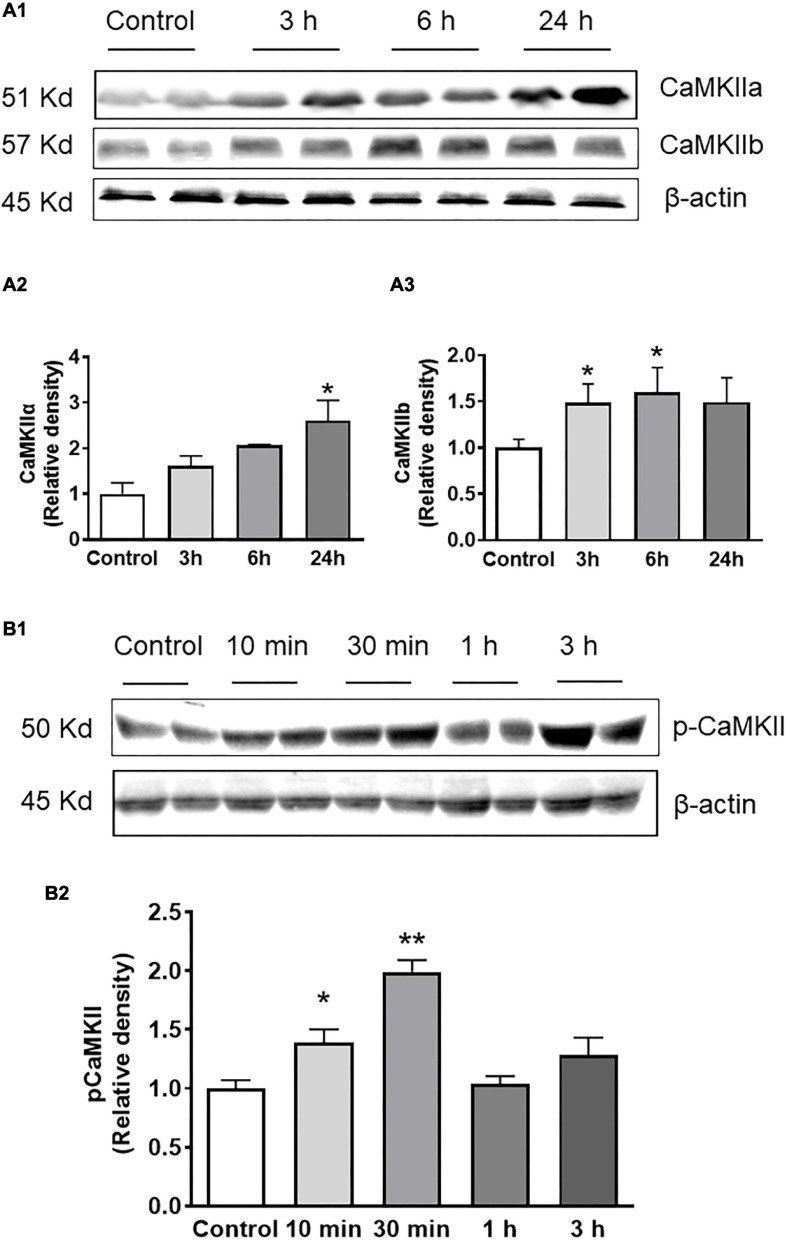
Orexin-A treatment increases CaMKII subunits’ protein expression as well as CaMKII activation in PC12-OX1R cells. PC12-OX1R cells were incubated with 100 nM orexin-A for the indicated periods. Proteins were separated and probed with CaMKIIa, CaMKIIb, P-CamKII, and β-actin antibodies. **(A)** Orexin-A treatment increases CaMKII protein expression in a time-dependent manner. **(A1)** Representative western blots showing CaMKIIa, CaMKIIb, and β-actin in orexin-A treated and control cells. Quantification of CaMKIIa **(A2)** and CaMKIIb **(A3)** that has been normalized against β-actin and compared with control, normalized to unity (*n* = 6, *P < 0.05). **(B)** Orexin-A treatment results in a time-dependent increase in P-CaMKII level. **(B1)** representative western blots showing P-CaMKII and β-actin. **(B2)** Quantification of P-CaMKII that has been normalized against β-actin and compared with control, normalized to unity (*n* = 6, *P < 0.05, **P < 0.01).

Consistent with real-time PCR data, western blot results showed that orexin-A treatment resulted in a time-dependent increase in CaMKIIa and CaMKIIb protein levels, with the maximum increase occurring 24 h following orexin-A treatment in CaMKIIa (2.6-fold) and 6 h after orexin-A treatment in CaMKIIb (1.6-fold) (Figure 4A). Furthermore, treatment of PC12-OX1R cells with orexin-A (100nM) resulted in a time-dependent increase in P-CaMKII, the activated form of the enzyme. The increase in P-CaMKII began approximately 10 min following orexin-A treatment, and reached a peak 30 min following treatment. 1 h following orexin-A incubation, basal levels of P-CaMKII were once again observed (Figure 4B).

### Orexin-A Treatment Increases P-CaMKII in Hypothalamic Neurons

**FIGURE 5:**
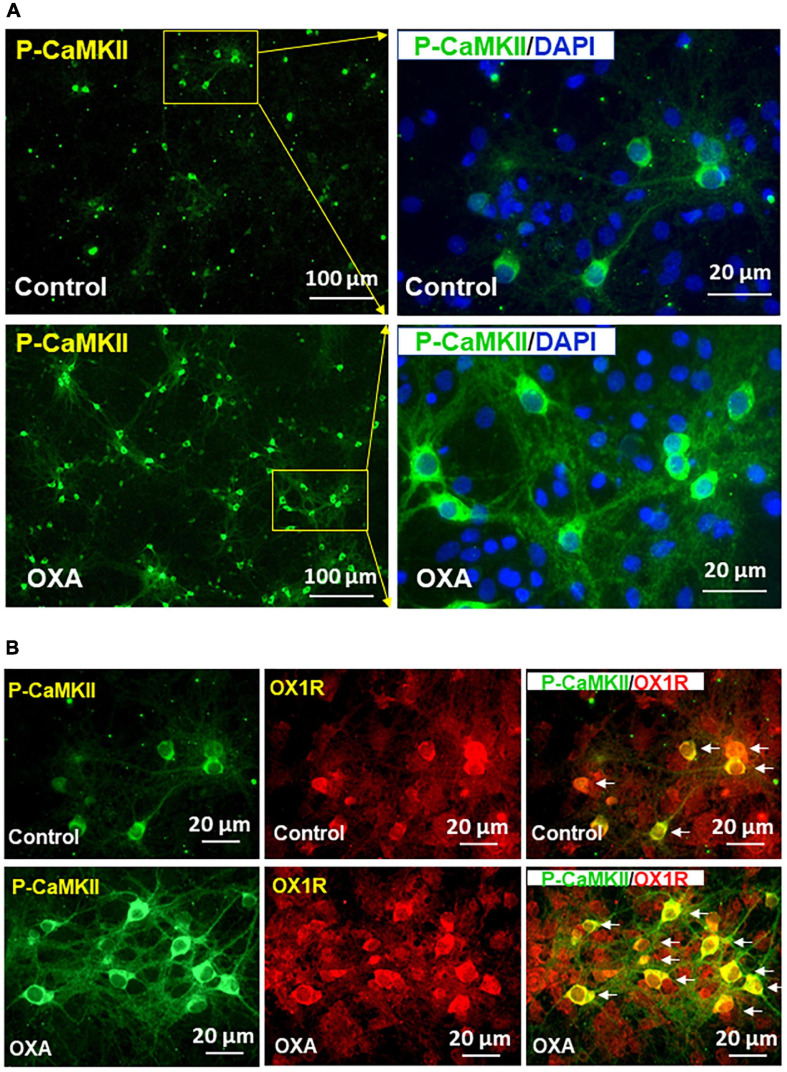
Orexin-A treatment increases CaMKII activation in OX1R-expressing hypothalamic neurons. Hypothalamic neuronal cultures isolated from neonatal SD rats were treated with 1 μM of orexin-A or vehicle control for 15 min, then cells were subjected to immunostaining using primary antibody anti-phospho-CaMKII (P-CaMKII) or OX1R followed by incubation with fluorescent dye-labeled secondary antibody. **(A)** Orexin-A treatment increases P-CaMKII in hypothalamic neurons. The area labeled in a solid rectangle in the left panel was higher magnified in the right panel. **(B)** Representative images show the immunostaining of P-CaMKII (green), OX1R (red), and merged image in cultured PVN neurons under orexin-A treatment.

Previous studies demonstrate that orexin-A can increase intracellular free [Ca^2+^] through binding with OX1R. Increased cytosolic [Ca^2+^] is known to be able to induce CaMKII autophosphorylation, therefore activating its kinase activity. In this experiment, we investigated whether orexin-A treatment can activate CaMKII in hypothalamic neurons (i.e., increasing P-CaMKII). If so, we wished to observe whether orexin-A induced CaMKII activation is mediated by OX1R activation. The result shows that orexin-A treatment for 15 min significantly increases P-CaMKII in hypothalamic neurons (Figure 5A). Co-immunostaining of P-CaMKII and OX1R demonstrated that CaMKII phosphorylation only occurs in OX1R expressing neurons (Figure 5B).

### Optogenetic Stimulation of PVN CaMKII-Expressing Neurons Causes Intensity-and Frequency-Dependent Increase in RSNA and Blood Pressure

**FIGURE 6:**
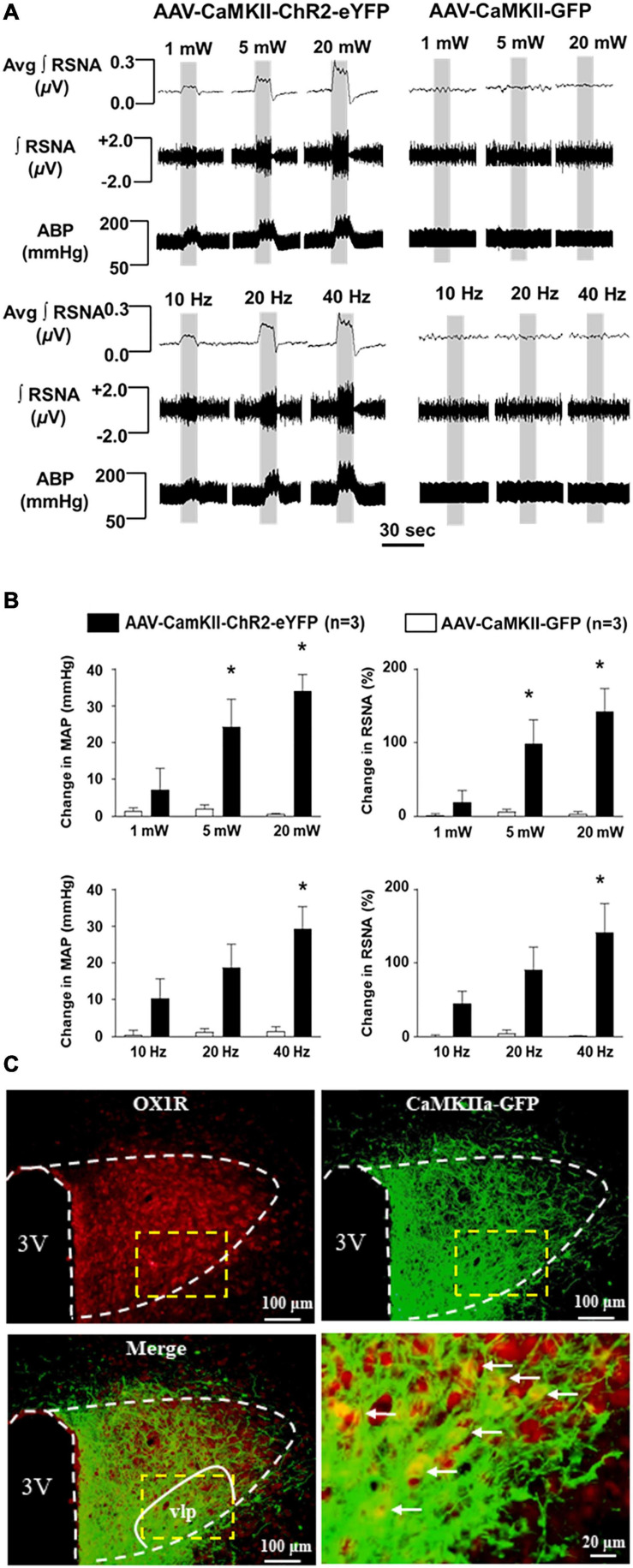
Optogenetic stimulation of CaMKII-expressing neurons in the PVN increases renal sympathetic nerve activity (RSNA) and arterial blood pressure (ABP). **(A)** Representative traces of RSNA and ABP during 10 sec optogenetic stimulation in the PVN at the intensity of 1, 5, and 20 mW (5 ms pulse duration) in AAV-CaMKII-ChR2-eYFP (left) and control vector AAV-GFP (right) injected rats (Top panels); and representative traces of RSNA and ABP during 10 sec optogenetic stimulation in the PVN at the frequency of 10, 20, and 40 Hz (5-ms pulse duration and 20 mW) in AAV-CaMKII-ChR2-eYFP (left) and AAV-GFP (right) injected rats (Low panel). **(B)** Population data showing the changes in RSNA and ABP in response to optogenetic stimulation of PVN at different intensities (top)- and frequencies (low). *P < 0.05, AAV-CaMKII-ChR2-eYFP vs AAV-CaMKII-GFP. Avg, average; ∫, integrated. **(C)** Immunostaining shows that PVN OX1R (red) and CaMKII (represented by report gene GFP) are co-localized in the PVN neurons, in particular, in the ventrolateral parvocellular (vlp) subdivision, a subdivision in the PVN rich in pre-sympathetic neurons (Stocker et al., 2004). 3V, the third ventricle. Low panel right is a magnified image of dashed line labeled area of the merged image (low panel, left).

To test the effect of PVN CaMKII expressing neurons on BP and sympathetic outflow, we injected viral vector AAV-CaMKII-ChR2-eYFP or control vector AAV-GFP viral vector into the PVN of adult SD rats. ChR2 (channelrhodopsin-2) is a direct light-gated cation-selective membrane channel driven by the CaMKII promoter and therefore is exclusively expressed in CaMKII-expressing neurons (Nagel et al., 2003). Optogenetic stimulation of the PVN neurons showed dramatic increases in RSNA and BP in an intensity-dependent manner in AAV-CaMKII-ChR2 injection rats. The maximum increase in RSNA (ChR2: 142.0 ± 31.7% vs. GFP: 3.1 ± 3.2%; *P* < 0.05) and mean arterial pressure (MAP) (ChR2: 34 ± 4.5 vs. GFP: 0.6 ± 0.4, mmHg; *P* < 0.05) occurred at the intensity of 20 mW, while no changes were observed in control viral vector, AAV-GFP injected rats. We further performed optogenetic stimulation of PVN neurons using different frequencies including 10, 20, and 40 Hz with 20 mW end output, respectively. It showed a frequency-dependent increase in RSNA and BP in AAV-CaMKII-ChR2 injection rats with the maximum increases in RSNA (ChR2: 141 ± 39.2% vs. GFP: 1.4 ± 1.3%, *P* < 0.05) and MAP (ChR2: 29.2 ± 6.1 vs. GFP: 1.4 ± 1.3 mmHg, *P* < 0.05) occurring at a frequency of 40 Hz. Again, no changes were observed in control viral vector injection rats (Figures 6A,B). This result demonstrated that excitation of PVN CaMKII expressing neurons increase SNA and MAP. Additionally, immunostaining of OX1R showed that OX1R is co-localized with GFP which was only expressed in CaMKII-expressing neurons. In particular, this co-localization was observed within the ventrolateral parvocellular (vlp) subdivision of the PVN, a brain area rich in pre-sympathetic neurons (Figure 6C; Stocker et al., 2004) suggesting that PVN OX1R is expressed in the CaMKII-expressing neurons.

## Discussion

This study is the first to investigate the contribution of the orexin system on the regulation of CaMKII, a key protein kinase involved in multiple functions including cardiovascular function and BP regulation (Mohler and Hund, 2011; Prasad et al., 2015). We report five major findings: (1) Orexin-A ICV injections elicit increased CaMKII mRNA expression in the PVN of SD rats; (2) OX1R is primarily expressed in neurons and astrocytes within the PVN; (3) Orexin-A treatment in PC12-OX1R cells results in increased CaMKII expression and activation, but this was not observed in PC12 cells; (4) Orexin-A treatment in hypothalamic neurons results in an increase in P-CaMKII, the activated form of the enzyme; and (5) Optogenetic stimulation of PVN CaMKII-expressing neurons results in increased RSNA and BP. Our results suggest that activation of orexin system activity in the PVN stimulates activation of the CaMKII pathway. This activation may subsequently facilitate, to a certain degree, the pressor effect observed by optogenetic stimulation of PVN CaMKII expressing neurons, although this effect has not yet been established.

Orexin system dysfunction, specifically hyperactivity, has been implicated in numerous experimental hypertensive animal models including the Obese Zucker rats (Zhou et al., 2015, 2019b), spontaneously hypertensive rats (Li and Nattie, 2014; Lee et al., 2015), and Dahl salt-sensitive rats (Huber et al., 2017). Furthermore, blockage of orexin receptors in the PVN has been observed to attenuate hypertension development (Carrive, 2013; Zhou et al., 2019a). However, the detailed mechanism mediating the effect of increased orexin system activity on hypertension development is not clear.

Previous studies show that binding of orexin-A with its receptors can cause calcium ion influx into the cytosol, increasing intracellular calcium concentrations (Holmqvist et al., 2002; Kohlmeier et al., 2008; Xia et al., 2009). However, the downstream signaling cascade components triggered by increased intracellular calcium is not clear. Calcium is a very important second messenger and can bind to calcium calmodulin protein, leading to CaMKII autophosphorylation, therefore activating its kinase activity (Wei et al., 2017). Interestingly, Li et al. (2017) observed that CaMKII hyperactivity within the PVN modulates the activity of the N-methyl-D-aspartate receptor, resulting in increased sympathetic outflow. However, the cause of this increased CaMKII activity has not been established. Given the similarly elevated activity of the orexin system and CaMKII, as well as the similar resultant increases in sympathetic activity and BP in hypertensive animal models (Li et al., 2017; Zhou et al., 2019a), we hypothesized that orexin signaling may lie upstream from CaMKII activation.

The present study shows that ICV administration of orexin-A into SD rats triggers modest yet significant increase in mRNA expression of CaMKII subunits, CaMKIIa and CaMKIIb, in the PVN (Figure 1), indicating a causative relationship between orexin-A release and CaMKII expression. This observation coupled with the evidence that ICV injection of orexin-A increases BP and sympathetic outflow in conscious SD rats (Samson et al., 1999; Shirasaka et al., 1999) suggests that hyperactivity of brain orexin system may, through stimulation of PVN CaMKII expression, result in the previously observed increases in SNA and BP. However, we are careful to note that this hypothesis requires further testing. The reasons for the observed moderate instead of high increase in CaMKII in response to ICV orexin infusion have several possible explanations: First, CaMKII and OX1R are expressed in the same cells in the PVN. Upon binding of orexin-A and OX1R, the OX1R-expressing cells undergo excitation which in turn elicits CaMKII expression. This process only occurs in OX1R-positive cells. PVN OX1R is primarily distributed in neurons as well as some astrocytes, as opposed to microglial cells (Figure 2). Therefore, testing CaMKII levels using whole PVN tissue samples, which also contain many OX1R-negative cells, will dilute the actual CaMKII increase which is reflected in our observations. Second, it is quite possible that central ICV administration of orexin-A activates OX1R distributed in other brain areas which through axonal projections synapse with CaMKII-expressing neurons in the PVN, impacting the observed expression levels. Third, in order to minimize animal use, we tested CaMKII levels 3 h after Orexin-A microinjection. However, it is worth noting that maximum CaMKII increases may occur before or after 3 h post orexin-A administration.

The stimulation effect of orexin-A on CaMKII expression shown in ICV injection is strengthened by a time-dependent increase in CaMKIIa and CaMKIIb mRNA and protein expression in PC12-OX1R cells, but not in PC12 cells which lack OX1R expression (Figures 3A,B), following orexin-A incubation. This suggests selective stimulation of CaMKII expression, specifically through downstream signaling associated with OX1R and orexin-A binding. Interestingly, the increase in CaMKIIa and CaMKIIb in PC12-OX1R was blocked by pre-incubation of the cells with EGTA-AM (Figure 3C) which chelate calcium ions (divalent cation), suggesting that increased intracellular calcium ions due to OX1R activation mediate the increase in CaMKIIa and CaMKIIb expression. This aligns with previous literature observing the direct impact of orexin signaling on increased calcium influx into the intracellular area and outlines CaMKII as a downstream target of this cation influx (Holmqvist et al., 2002). In addition to increased expression, western blot analysis following orexin-A treatment revealed increased phosphorylated CaMKII (P-CaMKII) expression, an active form of CaMKII enzyme, in PC12-OX1R cells (Figure 4). The combination of these results suggests that OX1R activation can increase CaMKII expression and CaMKII activation.

While PC12 cells offer some mechanistic insight, their physiological relevance may be questioned given that they are not brain neurons. However, we observed similar results in hypothalamic neuronal cells. P-CaMKII was significantly elevated in response to orexin-A incubation (Figure 5), and the P-CAMKII was exclusively shown in the OX1R-expressing neurons, strengthening the role of orexin-A-OX1R binding in CaMKII activation.

Lastly, our study shows that optogenetic stimulation of CaMKII neurons resulted in drastic increases in RSNA and BP (Figures 6A,B), replicating previous findings (Li et al., 2017). While this does not specifically indicate a direct role for orexin stimulation as a mediator of this process, it does show that PVN activation of CaMKII leads to a sympathetic pressor response. Since ICV injection of orexin-A into SD rats can increase their BP and RSNA (Samson et al., 1999; Shirasaka et al., 1999), upregulate PVN CaMKII expression (Figure 1), and excitation of PVN CaMKII-expressing neurons can increase their sympathetic outflow and BP (Figure 6), we speculate that increased orexin signaling may, through CaMKII activation, result in increased sympathetic outflow and BP, complimenting prior findings (Li et al., 2017). However, further studies regarding this mechanism are necessary to assess whether increased BP and SNA are blocked in response to central orexin-A administration following simultaneous Orexin-A microinjection and CaMKII inhibition. Li et al. (2017) showed that inhibition of CaMKII activity within the PVN using autocamtide-2-related inhibitory peptide resulted in a significant reduction of both ABP and sympathetic outflow. However, the effect of CaMKII inhibition on Orexin-A mediated sympathetic and pressor responses remains yet to be elucidated, although this outlines an exciting new avenue for future research. Until tested, the relationship between the observed influence of Orexin-A on CaMKII expression and its potential subsequent impact on cardiovascular control remains speculative.

We acknowledge that the current study has some limitations. First, as mentioned earlier, to confirm that the increased SNA and pressor response induced by upregulated orexin signaling is mediated by CaMKII activation, a CaMKII inhibitor needs to be administered to test whether the increase in BP and RSNA induced by ICV orexin-A microinjection can be abolished or attenuated. Second, although orexin-A treatment results in CaMKII activation in OX1R-expressing neurons in hypothalamic primary neuronal cultures (Figure 5), CaMKII may also be expressed in non-OX1R-expressing cells in the PVN. This is particularly relevant due to the evidence showing that GFP expression driven by the CaMKII promoter is not limited to OX1R immunoreactive cells (Figure 6C). Therefore, the pressor response observed following CaMKII excitation may be a combined effect of multiple cells including OX1R-expressing and OX1R-negative neurons. Third, increased orexin signaling may trigger other mechanisms such as neuronal hormone secretion, in turn regulating BP further. This may be in part due to the expansive PVN OX1R expression in not only pre-synaptic neuronal areas, but also areas involved in neurosecretory processes as suggested by the prominent distribution of OX1R in the magnocellular region of the PVN (Figure 6C). With regard to the third possibility, recent studies from our group suggest that activation of PVN OX1R can also increase PVN vasopressin expression (Huber et al., 2017; Bigalke et al., 2021). The current research study does not allow us to elucidate the vast number of possible molecular mediators at play, although these limitations offer future directions for exploration.

**FIGURE 7:**
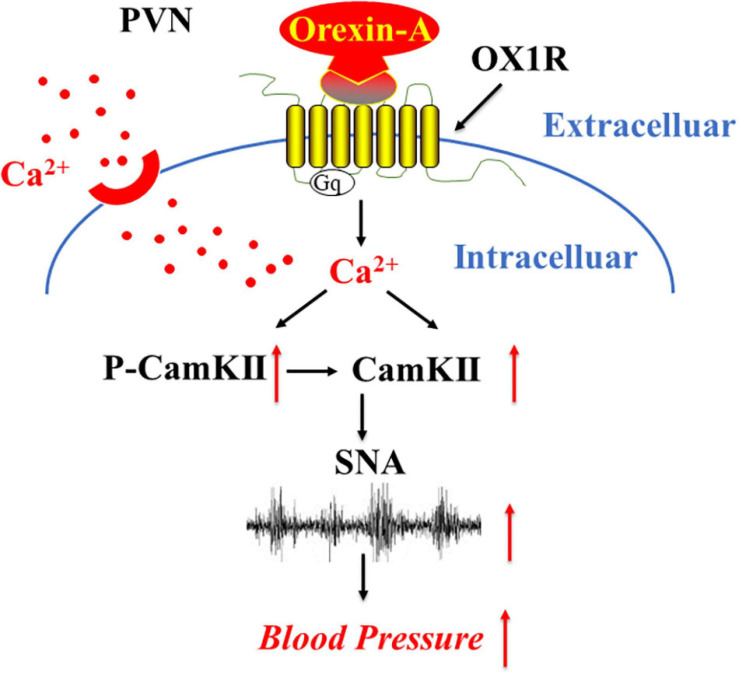
A proposed mechanism that OX1R activation on sympathetic nerve activity and blood pressure regulation. Binding of orexin-A with OX1R results in OX1R activation, which in turn triggers the influx of Ca^2+^ resulting in intracellular Ca^2+^ increase. Ca^2+^, in turn, activates CaMKII enzyme activity as well as CaMKII expression, which eventually results in SNA increase and blood pressure elevation.

Calmodulin-dependent kinase II has been shown in recent years to have an impact on the development of hypertension (Zhuang et al., 2016; Li et al., 2017). Our results offer insight into the upstream activity of the orexin system as a potential mediator of the deleterious impact of CaMKII in the development of hypertension, through excessive CaMKII activation. In conjunction with the results of Li and colleagues (Li et al., 2017), our proposed mechanism utilizes a feed-forward circuit, whereby orexin overactivity results in calcium influx, which activates CaMKII, resulting in modulation of pre-sympathetic neuronal firing patterns (Figure 7). Furthermore, these results strengthen the hypothesized role of the orexin system in BP and cardiovascular regulation. Our study suggests that increased orexin system activity may lead to an increase in CaMKII expression and excitation of CaMKII positive PVN neurons. Whether this over-abundance of orexin mediated CaMKII activity results in increased SNA and BP remains unknown, and is a target for future studies. This pathway offers a pertinent target for future research and warrants further investigation utilizing hypertensive animal models.

## Data Availability Statement

The original contributions presented in the study are included in the article/supplementary material, further inquiries can be directed to the corresponding author.

## Ethics Statement

The animal study was reviewed and approved by Michigan Technological University Institutional Animal Care and Use Committee (IUCAC).

## Author Contributions

YF, QC, and ZS conceived the research study, and analyzed and interpreted the results. YF, EJ, HG, JB, QC, CY, and ZS carried out all research protocols. BC involved in data discussion. YF, EJ, JB, QC, and ZS prepared and reviewed the final manuscript. All authors contributed to the article and approved the submitted version.

## Conflict of Interest

The authors declare that the research was conducted in the absence of any commercial or financial relationships that could be construed as a potential conflict of interest.

## Footnotes

1 http://rsb.info.nih.gov/ij
